# Supplementary material for: Oil palm expansion increases the vectorial capacity of dengue vectors in Malaysian Borneo
Source: PLoS Negl Trop Dis. 2022 Mar 16;16(3):e0009525. doi: 10.1371/journal.pntd.0009525 (PMC8959159; doi:10.1371/journal.pntd.0009525)
Supplement: S1 Methods — (DOCX) [file pntd.0009525.s001.docx]

S1 Methods

Human landing catch site selection:

A pilot survey of sites for adult mosquito collections was conducted at the four available human settlements in logged forest (A, B) and oil palm plantation (C, D), and at four randomly selected larval sampling sites in continuous forest and plantation over the course of two weeks in January each year. The forest and plantation sites were approximately 20km apart, and the distance between sites in each land-use was approximately 2km. Initial human landing catch attempts in continuous habitat sites did not yield any *Ae. albopictus* mosquitoes, so sampling efforts were focused on the four available human settlements logistically available for frequent sampling. Both oil palm sites were located in the Selangan Batu estate, a bench-terraced plantation with a dense network of access roads. The estate has a number of small housing clusters where plantation workers and their families reside, as well as administrative offices, a guesthouse, a school and supply shops. Site A was located next to the Selangan Batu guesthouse (4.6460 ̊N, 117.4514 ̊E), which is an exposed site surrounded by mature oil palms (planted in 2006). Site B was located outside a small roadside supply shop (4.64005 ̊N, 117.4491 ̊E). The site was also relatively exposed and surrounded by mature oil palm. Site C was the SAFE project research camp (4.7225 ̊N, 117.6003 ̊E**)**, which is a small cleared area with multiple bungalows, surrounded by logged forest. Site D was located at an active sawmill (4.6409 ̊N, 117.5827 ̊E); a large clearing separated into a sawmill area near the adjoining road, and worker residences closer to surrounding logged forest. Adult sampling occurred near the residences. No data on human density at these sites was available, however based on observation and the presence of housing, density was likely to be highest at Sites A and C.
