# Supplementary material for: Oil palm expansion increases the vectorial capacity of dengue vectors in Malaysian Borneo
Source: PLoS Negl Trop Dis. 2022 Mar 16;16(3):e0009525. doi: 10.1371/journal.pntd.0009525 (PMC8959159; doi:10.1371/journal.pntd.0009525)
Supplement: S2 Methods — (DOCX) [file pntd.0009525.s002.docx]

S2 Methods

Laboratory rearing protocol

The *Ae. albopictus* mosquitoes used were obtained from a colony of mosquitoes collected in New York State, USA. Adult females were provisioned with defibrinated horse blood every 3-4 days using an artificial feeder (Hemotek Discovery Workshops, Accrington, UK) and provided with substrate for egg laying. Eggs between 2-4 weeks old were vacuum hatched in dechlorinated water over a period of 36 hours. First larval instars were reared at a density of 50 larvae per cup and provisioned with five pellets of fish food **(***Tetra Cichlid Colour*TM**)** daily. Larval rearing cups were placed in one of two incubators (Panasonic MLR-350H, temperature accuracy ±0.5 ̊C, RH accuracy ±0.5%) running fluctuating temperature treatments simulating mean hourly diurnal temperatures in either logged forest or oil palm plantation sampling sites (Supplementary T2). Relative humidity was kept constant at 85%. Upon emergence, adults were provisioned with 10% sugar solution *ad libitum* and females offered defibrinated horse blood via

a Hemotek feeding system three days after emergence. Mosquitoes that did not fully imbibe were removed from the fecundity experiments. Mosquitoes that failed to feed were offered a bloodmeal each day until a successful feed was achieved or until four additional days had passed. Blood-fed females were transferred into individual laying tubes (2.9cm x 11.7cm) containing damp filter paper as an egg laying substrate. Papers were observed every day for the presence of eggs and the length of the pre-bloodmeal period and first gonotrophic cycle recorded.
