# Supplementary material for: Oil palm expansion increases the vectorial capacity of dengue vectors in Malaysian Borneo
Source: PLoS Negl Trop Dis. 2022 Mar 16;16(3):e0009525. doi: 10.1371/journal.pntd.0009525 (PMC8959159; doi:10.1371/journal.pntd.0009525)
Supplement: S1 Table — Temperatures were set to change every hour. Minimum (*) and maximum (Δ) temperatures for each treatment are indicated once in each column. (DOCX) [file pntd.0009525.s003.docx]

**S1 Table. Temperature schedules of incubators used for larval rearing.** Temperatures were set to change every hour. Minimum (*) and maximum (_∆_) temperatures for each treatment are indicated once in each column.

| Time (hours) | Logged forest incubator (°C) | Oil palm incubator (°C) |
| --- | --- | --- |
| 0 | 23 | 23 |
| 1 | 23 | 23 |
| 2 | 22* | 22* |
| 3 | 22 | 22 |
| 4 | 22 | 22 |
| 5 | 22 | 22 |
| 6 | 22 | 22 |
| 7 | 22 | 23 |
| 8 | 23 | 25 |
| 9 | 24 | 27 |
| 10 | 25 | 27 |
| 11 | 26 | 28 |
| 12 | 27 _∆_ | 29_∆_ |
| 13 | 27 | 29 |
| 14 | 27 | 29 |
| 15 | 27 | 28 |
| 16 | 27 | 27 |
| 17 | 26 | 26 |
| 18 | 25 | 25 |
| 19 | 24 | 24 |
| 20 | 24 | 24 |
| 21 | 23 | 23 |
| 22 | 23 | 23 |
| 23 | 23 | 23 |
