# Supplementary material for: Oil palm expansion increases the vectorial capacity of dengue vectors in Malaysian Borneo
Source: PLoS Negl Trop Dis. 2022 Mar 16;16(3):e0009525. doi: 10.1371/journal.pntd.0009525 (PMC8959159; doi:10.1371/journal.pntd.0009525)
Supplement: Attachment — Submitted filename: Review of albopicxtus palm oil.pdf [file pntd.0009525.s005.pdf]

Review of: Oil palm expansion increases the vectorial capacity of dengue vectors in Malaysian Borneo

By Gregory et al.

This is a carefully written and analyzed contribution to the literature aimed at understanding the effect of land use changes (and implicitly climate change) on the vectorial capacity of mosquitoes, in this case *Aedes albopictus.* The paper is a welcome contribution to a finer-scale understanding of LULC effects on mosquito vector capacity.

I have made numerous notes and style suggestions in the marked-up PDF. When it was an obvious style error, I simply highlighted the text.

Throughout, I was confused regarding the source of data that was used for analysis. There are numerous other citations including Mordecai but it was not clear from the text whether this manuscript describes analysis of a totally idependent data set or a re-analysis of the same data by the same team. This could be made much clearer, and where the data could have come from this report, why it was not used. I'm missing something here.

I have pointed out a terminology issue which is central to the significance and understanding of this paper. They authors use 'capacity' in a way that appears to be independent of density and may wish to reconsider their terminology. I have made notes on this in the marked-up PDF. The start of the problematic language is here.

"As mosquito density (m) scales proportionally with vectorial capacity, omitting abundance from the vectorial capacity equation allows us to compare the relative contributions of an individual mosquito inhabiting logged forest and oil palm plantation to transmission. We calculated this relative vectorial capacity (rVC, the vectorial capacity relative to the vector : human population ratio) for each of the sites."

It is possible the authors need to define the significance of the term more clearly or not use 'capacity' at all since that's a population term.

One of my main concerns is the sample size which seems quite small. From a total of 276 mosquitoes that were collected, many were males. While field data 'is what it is' and cannot be controlled, this seems to reduce the power of the analysis and should be discussed.

One variable that is important for mosquito density is the density of humans. While villages were compared, there is no description of the density of villages or humans over the study areas. Is it possible that human density due to villages and workers is a stronger correlate of mosquito density and is an unrecognized variable that is, or is not, associated with palm oil plantations? You might want to discuss this in the section starting with line 435.
