# Supplementary material for: Oil palm expansion increases the vectorial capacity of dengue vectors in Malaysian Borneo
Source: PLoS Negl Trop Dis. 2022 Mar 16;16(3):e0009525. doi: 10.1371/journal.pntd.0009525 (PMC8959159; doi:10.1371/journal.pntd.0009525)
Supplement: Attachment — Submitted filename: Authors_Response2.docx [file pntd.0009525.s006.docx]

21 October 2021

Dear Editors,

We are submitting revisions for “Oil palm expansion increases the vectorial capacity of dengue vectors in Malaysian Borneo” (Article) submitted for review by PLoS Neglected Tropical Diseases. All authors have approved the enclosed changes to the manuscript. We thank you and the reviewers for very useful comments that have strengthened the manuscript enormously. We have aimed to address all of the comments, and explain each of our responses (in italics) in detail below.

Thank you for your consideration once more. We look forward to your correspondence.

Sincerely,

Nichar Gregory

*Robert M. Ewers, Arthur Y.C. Chung, Lauren J. Cator*

**Response to Reviewer 1 Comments:**

Reviewer #1: This is an outstanding and important paper that combines field data, laboratory experiments, and mathematical models to mechanistically dissect the impact of palm oil plantations, relative to logged forests, on vectorial capacity of *Aedes albopictus* for arboviruses like dengue. I have relatively few minor comments on this overall excellent paper.

One important innovation of this study is that the authors estimated mortality rates and mosquito abundance directly in the field. These have previously been major unknowns because they were derived from laboratory estimates, and how well they applied to the field was uncertain.

The equation for m (eq. 2) uses the parameter EFD = eggs per female per day. However, in the original work characterizing the thermal performance curves (Mordecai et al. 2017), the trait estimated was TFD = eggs laid per female per gonotrophic cycle, so the substitution EFD = TFD*a (where a is 1/oviposition cycle length; see supplementary Table A) was made to calculate m for Aedes albopictus. Please note this, and correct the m calculation if needed.

- *The correct parameter metric was noted when originally estimating m, so no changes have been made. We have clarified this in the text (lines 262-263).*

Regarding the calculation of the mortality rate, mu, note that in the previous work that derived the thermal performance curves (Mordecai et al. 2017), mu was defined as a mortality rate (units of 1/day) rather than a probability, as stated in line 305. Based on the calculation of mu as 1/lifespan, I believe that the calculation was correct, just the description of the parameter as a probability is not. I didn’t understand how the calculation of 33.3 +/- 57 days was estimated, since if the mortality rate is 0.03 +/- 0.006, that gives the range in mortality rates of 0.036 (lifespan = 27.8 days) to 0.024 (lifespan = 41.7 days).

- *We have corrected the description of the parameter, mu, throughout the text. Thank you for highlighting the standard error typo! The lifespan should read 33.3 +/- 7 days, not 57 (line 353).*

In line 197 it says that temperature variation was higher in logged forest than in oil palm plantations, but in lines 316-317 it says the opposite. Which is correct? Even though temperature variation turned out to not play an important role in the mosquito traits, it is still important to clarify this point.

- *Thank you for catching this mistake – temperature variation was higher in oil palm plantations. We have amended the text accordingly (lines 230-231).*

Minor comments:
The Abstract and the Author Summary only mention *Aedes albopictus* and vectorial capacity, but not which diseases are actually at risk of being transmitted in this system. Especially if this study is motivated by an observation of increased disease incidence in people that contact plantations, it is worth mentioning what disease(s) are important here.

- *The abstract and author summary have been amended to include mention of the relevant arboviruses.*

Line 29: “predications” should be “predictions”

- *Word changed in text.*

Line 48: typo in “anthropogenic”

- *Word amended in text.*

There are several locations throughout the manuscript where the citations aren’t in the PLOS NTD numbered format.

- *Citation inconsistencies have been amended.*

Lines 141 and 495: “Aedes” should be italicized

- *Formatting amended in text.*

**Response to Reviewer 2 Comments:**

This is a carefully written and analyzed contribution to the literature aimed at understanding the effect of land use changes (and implicitly climate change) on the vectorial capacity of mosquitoes, in this case Aedes albopictus. The paper is a welcome contribution to a finer-scale understanding of LULC effects on mosquito vector capacity.

I have made numerous notes and style suggestions in the marked-up PDF. When it was an obvious style error, I simply highlighted the text.

Throughout, I was confused regarding the source of data that was used for analysis. There are numerous other citations including Mordecai but it was not clear from the text whether this manuscript describes analysis of a totally independent data set or a re-analysis of the same data by the same team. This could be made much clearer, and where the data could have come from this report, why it was not used. I’m missing something here.

- *We apologise for the confusion and have edited the text for clarity, including more ubiquitous reference to Fig 1, which illustrates the various sources of data used for each composite metric. We used our own data on mosquito density, survival and biting rate; when our own data were not used, it was either because we had not collected those data (e.g. REI or competence parameters) or because we were interested in comparing an entirely temperature-driven metric to field data (e.g. m). All temperature data used to derive thermal responses were collected by the same team and published in a previous manuscript (Gregory et al. 2019).*

I have pointed out a terminology issue which is central to the significance and understanding of this paper. They authors use ‘capacity’ in a way that appears to be independent of density and may wish to reconsider their terminology. I have made notes on this in the marked-up PDF. The start of the problematic language is here.

“As mosquito density (m) scales proportionally with vectorial capacity, omitting abundance from the vectorial capacity equation allows us to compare the relative contributions of an individual mosquito inhabiting logged forest and oil palm plantation to transmission. We calculated this relative vectorial capacity (rVC, the vectorial capacity relative to the vector : human population ratio) for each of the sites.”

It is possible the authors need to define the significance of the term more clearly or not use ‘capacity’ at all since that’s a population term.

- *We appreciate the reviewer pointing out this issue, and have endeavored to more clearly define the respective terms in the text (lines 278-280). We calculated both vectorial capacity (VC), which includes our density data, and relative vectorial capacity (rVC), which excludes the abundance parameter. We chose to include an estimate of rVC to account for the small sample size of our mosquito abundance data, which the reviewer correctly highlights in a later comment. Although rVC excludes density, it still incorporates extrinsic factors (e.g. mosquito mortality and biting rate), rendering it conceptually closer to vectorial capacity than to competence, which is governed by intrinsic (i.e. genetic) factors that influence the ability of a mosquito to transmit a pathogen. Omitting abundance from VC is a commonly used method to compare epidemic potential in space and time - we feel that we have used the terms appropriately and in a way that is consistent with the literature.*
- *Please find the following references that use rVC in this way:*
  - Liu-Helmersson, J., Stenlund, H., Wilder-Smith, A., & Rocklöv, J. (2014). Vectorial capacity of Aedes aegypti: effects of temperature and implications for global dengue epidemic potential. *PloS one*, *9*(3), e89783.
  - Shapiro, L. L., Whitehead, S. A., & Thomas, M. B. (2017). Quantifying the effects of temperature on mosquito and parasite traits that determine the transmission potential of human malaria. *PLoS biology*, *15*(10), e2003489.
  - Viennet, E., Mincham, G., Frentiu, F. D., Jansen, C. C., Montgomery, B. L., Harley, D., ... & Faddy, H. M. (2016). Epidemic potential for local transmission of Zika virus in 2015 and 2016 in Queensland, Australia. *PLoS currents*, *8*.

One of my main concerns is the sample size which seems quite small. From a total of 276 mosquitoes that were collected, many were males. While field data ‘is what it is’ and cannot be controlled, this seems to reduce the power of the analysis and should be discussed.

- *This is a valid point and motivated our calculation of relative vectorial capacity in addition to vectorial capacity. We have included acknowledgement of this limitation in the text (line 439).*

One variable that is important for mosquito density is the density of humans. While villages were compared, there is no description of the density of villages or humans over the study areas. Is it possible that human density due to villages and workers is a stronger correlate of mosquito density and is an unrecognized variable that is, or is not, associated with palm oil plantations? You might want to discuss this in the section starting with line 435.

- *This is an important point and we agree that variation in human density may impact mosquito density. We have expanded our discussion of the socio-economic and demographic factors that might mediate mosquito density at our sites (lines 498-504).*

Line 36: Meaning estimates of mosquito development related traits? Please clarify

- *Correct, we have edited the text for clarity.*

Line 111: Whilst this may be typical, I suggest you state what the best quality is that is available. Many readers will have no idea whether newer satellites offer higher resolution.

- *Reference to the best quality data available has been added to the text (line 111).*

Line 121: Throughout, the term 'forest conversion' is used. Does that term indicate a direction of conversion e.g. to grassland, urbanization etc?

- *In this study, forest conversion refers specifically to the conversion to agricultural land, specifically oil palm plantation. We have edited for clarity throughout the text.*

Line 125: Please insert a caveat if these outbreaks are caused by Aedes albopictus or aegypti. Might be a correlation without a causal link.

- *This is a good point – thank you. In Peninsular Malaysia, the vector species responsible for dengue outbreaks is suspected to be Ae. aegypti, however the dominant vector in Borneo has not yet been thoroughly investigated. Entomological surveys (e.g. Murphy* et al*. 2020; Rozilawati et al. 2015) have found that Ae. albopictus comprises the dominant - and sometimes only - vector species in dengue outbreak areas in Borneo. We have edited the text to include these details.*

Line 141: Conversation. Italics

- *Word changed to “conservation” in text and “Aedes” italicized in text and throughout manuscript.*

Line 194: Please be more specific about the volume of water in each cup. Even if it's an estimate.

- *The volume of water in each cup has been included in the text.*

Lines 197-198: Please specify whether the deviation actually resulted in a mean temperature of 24. That would mean e.g. the number of hours at +3 is equal to the number of hours -3. Right?

- *Thank you for highlighting this point – as we used the mean hourly temperature from the field, diurnal temperature cycles in the incubator were non-symmetrical resulting in slightly divergent mean temperatures (24.1˚C in logged forest and 24.8˚C in oil palm plantations). We have updated the text to reflect this and have included a table of the hourly incubator temperatures in the Supplementary methods (S2).*

Line 213: Suggest you specify 'wild adult mosquito density' and then drop 'adult' density in the next sentence.

- *Text has been changed as suggested.*

Line 220: Please specify the number of the reference. So is this the same data set being analyzed in this paper as Mordecai reported? If not, why did you not use your own data?*.*

- *The citation has been amended in the text. The datasets are different – the thermal response curves in Mordecai et al. (2017) were derived from mathematical models fit to data from various laboratory experiments not related to this study. We did not use our own data to derive density because we did not collect data on most underlying components of density (fecundity, larval mortality etc.). Additionally, we were interested in comparing an entirely temperature-derived metric to our observations in the field.*

Lines 229-230: Suggest you re-order the variables in the text to reflect the order they would be read in the formula, L to R, top to bottom.

- *The vectorial capacity parameters have been reordered as suggested.*

Line 241: This may be reasonable, however capacity is a *population* characteristic. If you remove abundance from the equation, isn't that closer to competence? Please discuss the relationship of these.

Whatever, this is, you need to justify how the use of the term capacity can be justified in the absence of abundance. Or are you saying, in addition to Vc as described above, you ALSO calculated the density independent factors?

When you are using either density dependent or independent terms must be crystal clear going forward. Otherwise, it's quite confusing.

For example, in section 2.6 you say survival strongly affects Vc. But would that not affect 'm'?

- *As stated above, we have used the terms in a manner that is consistent with the literature and feel strongly that the terminology should remain standardized.*
- *In Section 2.6, because we used field observations on density rather than deriving it from the underlying parameters, we did not incorporate feedbacks of changing mortality on m. We have included a note regarding this point (lines 401-403).*

Line 310: Suggest you add totals for each column to the table.

- *Totals have been added to the table as suggested.*

Line 317: Throughout, when you cite, specify the number of the reference regardless of whether you specify the authors. Again, here is an example where it is not clear whether you are referring to a different analysis of the same dataset that was published previously by some of the same authors. If there are such cases, they need to be described in the introduction or M & M or justified in some way.

- *Citations have been amended throughout.*

Line 321: Meaning from a single female? Not a population trait? Meaning a female that is infected? If it's infectious, why is the p not 1?

- *Yes, the parameter b describes the average probability of an individual infected mosquito from the population transmitting dengue to a susceptible human. Earlier derivations of vectorial capacity assumed that b = 1, however a growing body of laboratory data suggests that b is temperature-dependent and that that assumption is often violated. We elected to use an updated vectorial capacity metric that incorporates this insight.*

Line 457: As an adjective, this doesn't need to be capitalized.

- *Word corrected in text.*

Line 475: You may want to move this sentence to the section where I questioned the relevance of albopictus vs. aegypti.

- *As suggested, the relevance of Ae. albopictus for dengue transmission in the region has also been added to the introduction.*

Line 513: I did not check the style of the references but noted numerous instances in which genus-species was not italicized.

- *References have been checked and all genus-species have been italicized.*
